# A Novel Way of Preventing Postoperative Pancreatic Fistula by Directly Injecting Profibrogenic Materials into the Pancreatic Parenchyma

**DOI:** 10.3390/ijms21051759

**Published:** 2020-03-04

**Authors:** Sang Chul Lee, Tae Ho Hong, Ok-Hee Kim, Suk Joon Cho, Kee-Hwan Kim, Jin Sook Song, Kyu-Seok Hwang, Jae-Kyung Jung, Ha-Eun Hong, Haeyeon Seo, Ho Joong Choi, Joseph Ahn, Tae Yoon Lee, Eunyoung Rim, Kwan-Young Jung, Say-June Kim

**Affiliations:** 1 Department of Surgery, Daejeon St. Mary’s Hospital, College of Medicine, the Catholic University of Korea, Daejeon 34943, Korea; zambo9@catholic.ac.kr; 2 Catholic Central Laboratory of Surgery, Institute of Biomedical Industry, College of Medicine, The Catholic University of Korea, Seoul 06591, Korea; gshth@catholic.ac.kr (T.H.H.); ok6201@hanmail.net (O.-H.K.); keehwan@catholic.ac.kr (K.-H.K.); hhe49@naver.com (H.-E.H.); searcx12@naver.com (H.S.); 3 Department of Surgery, Seoul St. Mary’s Hospital, College of Medicine, The Catholic University of Korea, Seoul 06591, Korea; hopej0126@gmail.com (H.J.C.); josephmon@naver.com (J.A.); aroong318@gmail.com (T.Y.L.); 4 College of Pharmacy, Chungbuk National University, Cheongju 28644, Korea; seokjun@krict.re.kr (S.J.C.); orgjkjung@chungbuk.ac.kr (J.-K.J.); 5 Department of Surgery, Uijeongbu St. Mary’s Hospital, College of Medicine, The Catholic University of Korea, Seoul 11765, Korea; 6 Bio & Drug Discovery Division, Korea Research Institute of Chemical Technology, Daejeon 34114, Korea; jssong@krict.re.kr (J.S.S.); kshwang@krict.re.kr (K.-S.H.); krjeong@krict.re.kr (K.-Y.J.); 7 Deparpment of Medicinal Chemistry and Pharmacology, University of Science & Technology, Daejeon 34113, Korea; wydl3796@krict.re.kr

**Keywords:** fibrosis, pancreatic stellate cells, pancreas texture, penicillin G, postoperative pancreatic fistula (POPF), transforming growth factor-β1

## Abstract

This paper aims to validate if intrapancreatic injection of penicillin G can enhance hardness and suture holding capacity (SHC) of the pancreas through prompting the fibrosis process. Soft pancreatic texture is constantly mentioned as one of the most contributory predictors of postoperative pancreatic fistula (POPF). Soft pancreas has poor SHC and higher incidence of parenchymal tearing, frequently leading to POPF. From a library of 114 antibiotic compounds, we identified that penicillin G substantially enhanced pancreatic hardness and SHC in experimental mice. Specifically, we injected penicillin G directly into the pancreas. On determined dates, we measured the pancreatic hardness and SHC, respectively, and performed molecular and histological examinations for estimation of the degree of fibrosis. The intrapancreatic injection of penicillin G activated human pancreatic stellate cells (HPSCs) to produce various fibrotic materials such as transforming growth factor-β1 (TGF-β1) and metalloproteinases-2. The pancreatic hardness and SHC were increased to the maximum at the second day after injection and then it gradually subsided demonstrating its reversibility. Pretreatment of mice with SB431542, an inhibitor of the TGF-β1 receptor, before injecting penicillin G intrapancreatically, significantly abrogated the increase of both pancreatic hardness and SHC caused by penicillin G. This suggested that penicillin G promotes pancreatic fibrosis through the TGF-β1 signaling pathway. Intrapancreatic injection of penicillin G promotes pancreatic hardness and SHC by enhancing pancreatic fibrosis. We thus think that penicillin G could be utilized to prevent and minimize POPF, after validating its actual effectiveness and safety by further studies.

## 1. Introduction

Postoperative pancreatic fistula (POPF) is one of the most fatal complications following pancreatic surgery. According to the updated International Study Group of Pancreatic Fistula (ISGPF) definition in 2016, POPF is defined as a drain output in a measurable volume of fluid, on or after postoperative day (POD) 3, with an amylase value greater than three times the upper limit of serum amylase value, associated with a clinically relevant development/condition related directly to POPF [1]. Since pancreatic juice contains considerable amounts of digestive enzymes, which can dissolve surrounding tissues and even the blood vessels, POPF could threaten a patient’s survival. POPF is the cause of mortality in 0–5% of patients following a pancreatic surgery [2]. The incidence of POPF is about 5–30% in the recent literature [3,4,5,6,7,8,9,10]. Pancreatic surgery also has a high postoperative morbidity of up to 30–50% and POPF is the main reason for this morbidity [11]. POPF is associated with serious sequelae including sepsis, abscess, and hemorrhage, which are associated with prolonged hospital stays, increased healthcare costs, and possible mortality [9,12].

To reduce the incidence of POPF, investigators have attempted various strategies, including the refinement of surgical techniques [13,14,15,16], application of adhesive materials around pancreatic enteric anastomosis site [17,18] and intravenous administration of agents, which inhibit pancreatic secretion [19,20,21]. However, most of these attempts failed to show remarkable superiority in preventing POPF, ultimately leading to no significant reduction in the incidence of POPF over the last three decades [17,19,21,22,23,24,25,26,27,28]. We believe that the main reason for the failure of these approaches is that most of them do not target the root cause of pathogenesis in POPF.

Here, we propose a novel approach toward preventing POPF. There are disease-, patient-, and procedure-related factors for POPF [29,30]. Disease-related factors include pancreatic texture, pathology, duct size, and fat content. Patient-related factors include age, gender, malnutrition, and jaundice. Procedure-related factors include anastomotic technique, operation time, and intraoperative blood loss. Of them, numerous studies consistently indicated that pancreatic texture is the most contributory predictor of POPF [28,30,31,32]. Several meta-analyses reported that patients with soft pancreas have the significantly higher incidence of POPF than did those with hard pancreas [32,33]. Soft pancreas has poor suture holding capacity (SHC) and is frequently associated with parenchymal tearing arising from technical difficulties [31,34]. We herein focused on fibrosis, the essential component of natural wound healing processes, which is capable of making tissues harder and stiffer by accumulating collagens and other interstitial substances. Firstly, we attempted to find the compounds that could promote fibrosis of pancreatic tissue from an antibiotic drug library, and then to validate whether these compounds can enhance hardness and SHC of the pancreas by a series of in vitro and in vivo experiments.

## 2. Results

### 2.1. Selection of Pancreas-Hardening Compounds from Antibiotics Library

**Figure 1:**
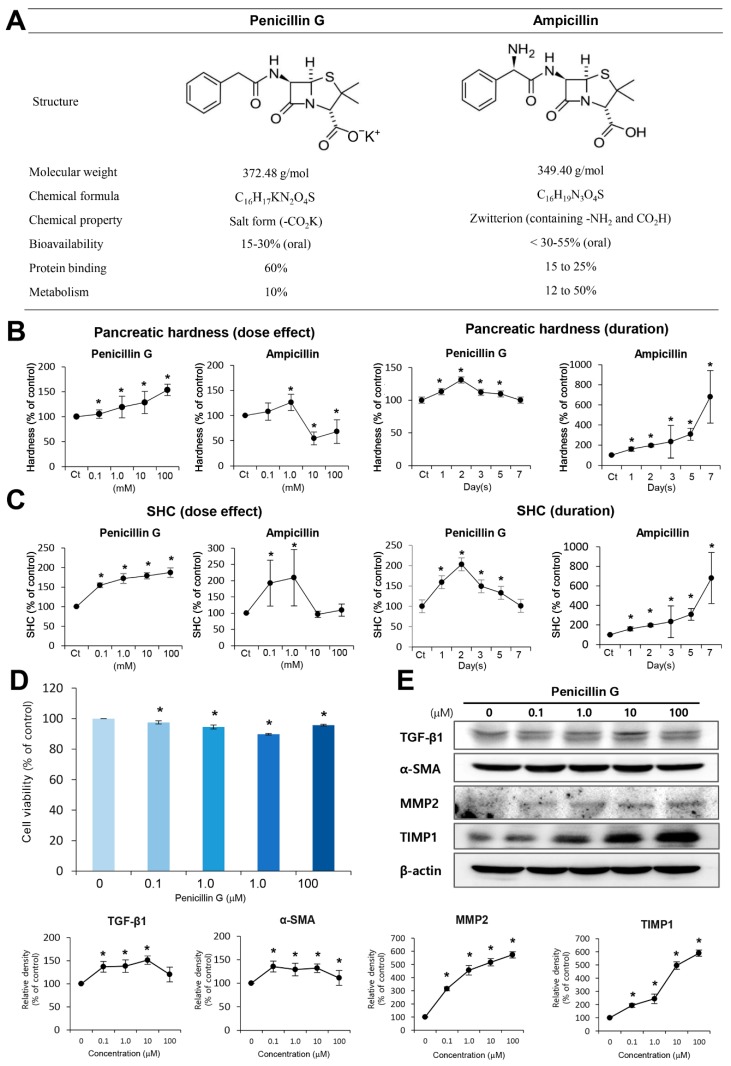
Comparison of pancreas-hardening capacity and SHC between penicillin G and ampicillin. (**A**) Molecular structure of penicillin G (Left) and ampicillin (Right). Ampicillin differs from penicillin G only by the presence of an amino group. (**B**) Pancreas-hardening capacity between penicillin G and ampicillin. [Top] Dose-dependent effects of penicillin G (Left) and ampicillin (Right) on hardening pancreas. Whereas penicillin G increased the pancreatic hardness dose-dependently, ampicillin showed the pattern of increasing the pancreatic hardness, especially at a certain concentration (1 mM). [Bottom] Alterations of pancreatic hardness over time between ampicillin-injected (Left) and penicillin G-injected (Right) mice. While the pancreatic hardness increased to the maximum at the second day in the penicillin G-injected mice, the hardness of the pancreas gradually increased over time until the seventh post-injection day in the ampicillin-injected mice. (**C**) Suture-holding capacity (SHC) of the pancreas between varying concentrations of penicillin G and ampicillin [Top] Dose-dependent effects of SHC of the pancreas between penicillin G [Left] and ampicillin [Right]. Whereas penicillin G increased the SHC of the pancreas dose-dependently, ampicillin increased it at certain concentrations (0.1 and 1.0 mM) only. [Bottom] Alterations of SHC of the pancreas over time between ampicillin-injected [Left] and penicillin G-injected [Right] mice. Whereas the SHC of the pancreas increased to the maximum at the second day after injection in the penicillin G-injected mice, it gradually increased over time until the seventh post-injection day in the ampicillin-injected mice. (**D**) Cell viability assay of penicillin G using human pancreatic stellate cells (HPSCs). Penicillin G did not decrease the viability of HPSCs within the tested concentrations (100 nM–100 μM) (**E**) Western blot analysis of HPSCs after treatment of penicillin G. Penicillin G increased the expression of fibrosis-related proteins, such as TGFβ-1, α-SMA, MMP2, and TIMP1, dose-dependently. Values are presented as mean ± standard deviation of three independent experiments. * *p* < 0.05. Abbreviations: α-SMA, alpha smooth muscle actin; DMSO, dimethyl sulfoxide; MMP-2, metalloproteinases-2; HPSCs, pancreatic stellate cells; SHC; suture-holding capacity; TGF-β1, transforming growth factor-β1; TIMP1, tissue inhibitor of metalloproteinases-1.

Pharmaceutical compounds can evoke fibrosis as a side effect. Since the safety of antibiotics has already been firmly established, we sought to find the compounds that enhance pancreatic hardness and SHC after direct injection into the pancreas. As a screening test to identify candidate antibiotics, we examined the changes of pancreatic hardness and SHC after direct injection of each of the antibiotic in pancreas of the experimental mice. From 114 numbers of compounds in the antibiotic library, we found that only ampicillin and penicillin G substantially enhanced pancreatic hardness and SHC of experimental mice (Figure 1A).

We further investigated how penicillin G and ampicillin affects pancreatic hardness and SHC with time and concentration. On day 2 post-injection, while penicillin G increased the pancreatic hardness dose-dependently, ampicillin showed a pattern of increasing the pancreatic hardness at a certain concentration only (1 mM) (Figure 1B Top). Next, we compared the alteration of pancreatic hardness over time between the two groups (Figure 1B Bottom). In the penicillin G-injected group, the pancreatic hardness increased to the maximum at the second day after injection, and then it subsided gradually, demonstrating the reversibility of the hardness induced by penicillin G.

Subsequently, we compared the SHC of mouse pancreas after treating them with different concentrations of both the antibiotics. While penicillin G increased the SHC of the pancreas dose-dependently, ampicillin increased it at certain concentrations (0.1 and 1.0 mM) at day 2 post-injection (Figure 1C Top). Next, we compared the alteration of SHC of the pancreas over time between the two groups (Figure 1C Bottom). In the penicillin G-infusion group, the SHC of the pancreas increased to the maximum at the second day after injection, and then it gradually subsided, demonstrating the reversibility of SHC induced by penicillin G. Ideal pancreas-hardening materials should have (1) consistent and high pancreas-hardening capacity and SHC, (2) the highest hardness on 2–3 days post-injection when the incidence of pancreatic fistula is peaked, and (3) the reversibility of fibrosis to avoid the persistent damage to the pancreas. Since we thought that penicillin G satisfies with these conditions, we conducted further studies using penicillin G.

### 2.2. In Vitro Determination of Profibrotic Property of Penicillin G

To determine cytotoxicity, we first performed cell viability test of penicillin G using HPSCs (Figure 1D). Cell viability test demonstrated no cytotoxicity by penicillin G within the tested concentration range (100–100 μM). Subsequently, after treating the HPSCs with penicillin G, we performed Western blot analysis to determine the expression patterns of proteins related to fibrosis, such as transforming growth factor-β1 (TGFβ-1), alpha smooth muscle actin (α-SMA), metalloproteinases-2 (MMP2), and tissue inhibitor of metalloproteinases-1 (TIMP1). Penicillin G increased the expression of these fibrosis-related proteins in a dose-dependent manner (Figure 1E).

### 2.3. In Vivo Determination of Profibrotic Property of Penicillin G

**Figure 2:**
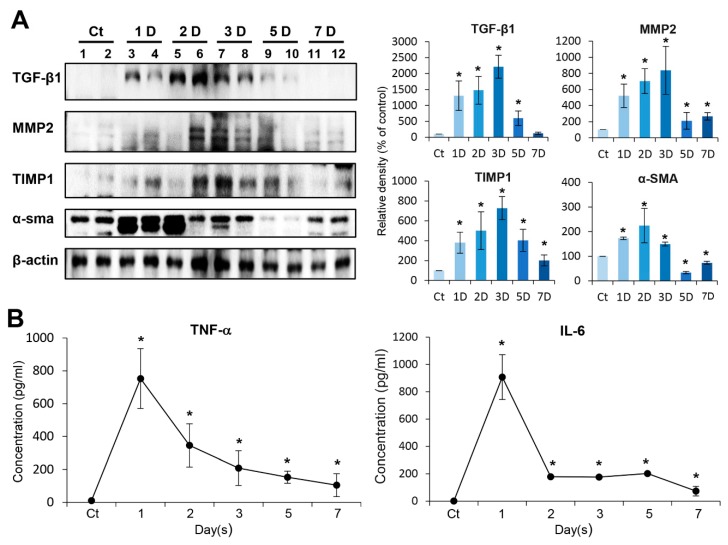
In vivo determination of profibrotic property of penicillin G. (**A**) Western blot analysis of mouse pancreas after direct injection of penicillin G into mouse pancreas over time. The expression of TGF-β1, MMP2, and TIMP1 in the pancreas gradually increased and peaked after three days of injection with penicillin, and then decreased gradually. The expression of α-SMA peaked at the second day after injection and gradually decreased thereafter. (**B**) Enzyme linked immunosorbent assay (ELISA) of serum pro-inflammatory cytokines (IL-6 and TNF-α) after injection of penicillin G over time. The levels of IL-6 and TNF-α peaked at day 1 post-injection with penicillin, and almost subsided by seven days post-injection. Values are presented as mean ± standard deviation of three independent experiments. * *p* < 0.05. Abbreviations: α-SMA, alpha smooth muscle actin; MMP-2, metalloproteinases-2; TGF-β1, transforming growth factor-β1, TIMP-1, tissue inhibitor of metalloproteinases-1, TNF-α, tumor necrosis factor-α.

We intended to determine day-by-day changes of the expression of fibrosis-related proteins (TGF-β1, MMP2, TIMP1, and α-SMA) in the pancreas after intra-pancreatic injection of penicillin G using Western blot analysis (Figure 2A). The expression of TGF-β1, MMP2, and TIMP1 in the pancreas gradually increased after injection and peaked at day 3 after injection and then gradually decreased. The expression of α-SMA peaked at the second day after injection and gradually decreased thereafter. We next investigated the serum levels of pro-inflammatory mediators, such as TNF-α and IL-6, after injecting penicillin G into the pancreas. The serum levels of these cytokines peaked during the first day after infusion, and then gradually decreased almost to normal levels by day 7 (Figure 2B).

### 2.4. Histological Changes Induced by the Injection of Penicillin G

**Figure 3:**
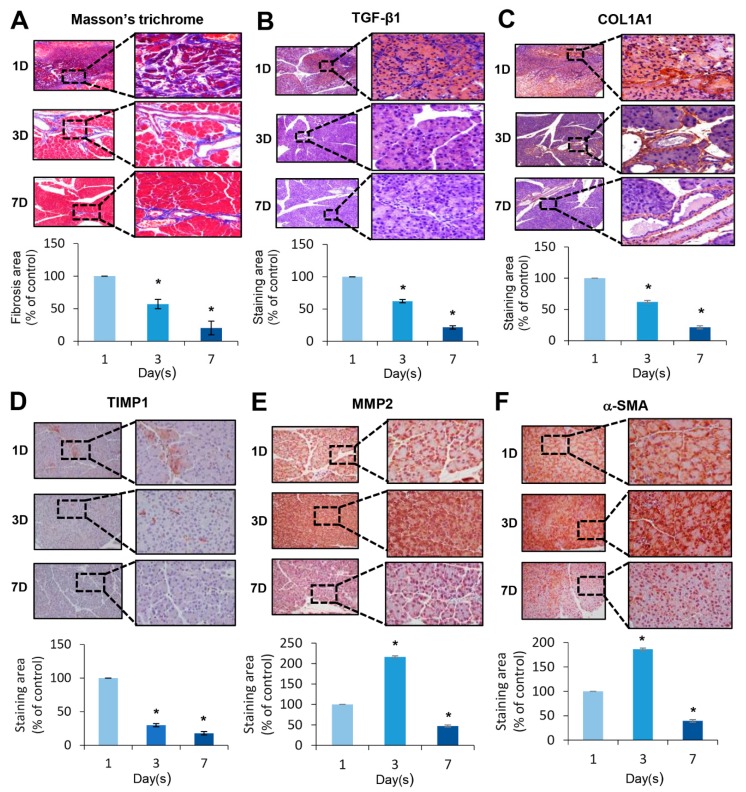
Histological changes induced by the injection of penicillin G. (**A**) Masson-trichrome stains of mouse pancreas after injection of penicillin G. The degree of fibrosis was highest at the first day after injection and decreased gradually thereafter until seven days after injection. (**B**–**F**) TGF-β1, COL1A1, TIMP1, MMP2, and α-SMA immunohistochemistry of mouse pancreas after injecting penicillin G. In TGF-β1, COL1A1, and TIMP1 immunohistochemistry, the expression of the markers was the highest on the first day after injection, and gradually decreased thereafter until seven days after injection. In MMP2 and α-SMA immunohistochemistry, the expression of the markers was highest at three days after injection and decreased thereafter until seven days after injection. Values are presented as mean ± standard deviation of three independent experiments. * *p* < 0.05. Abbreviations: α-SMA, alpha smooth muscle actin; COL1A1, collagen type 1 alpha 1; MMP-2, metalloproteinases-2; TGF-β1, transforming growth factor-β1, TIMP-1, tissue inhibitor of metalloproteinases-1.

To determine the extent of fibrosis, we performed Masson’s trichrome stain of the pancreas (Figure 3A). Masson’s trichrome stain showed that the degree of fibrosis on the pancreas were highest at the first day after injection, gradually decreasing thereafter until seven days after injection. For the precise determination of fibrosis, we subsequently performed IHC studies on the pancreas using antibodies reflecting fibrosis, such as α-SMA, MMP2, TGF-β1, and TIMP1, on post-injection day 1, 3, and 7 after injection (Figure 3B–F). In TGF-β1, COL1A1, and TIMP1 immunohistochemistry, the expression of the markers was the highest on the first day after injection, and gradually decreased thereafter until seven days after injection. In MMP2 and α-SMA immunohistochemistry, the expression of the markers was highest at the third day after injection and decreased thereafter.

### 2.5. Effects of Penicillin G on the Pancreatic Function

**Figure 4:**
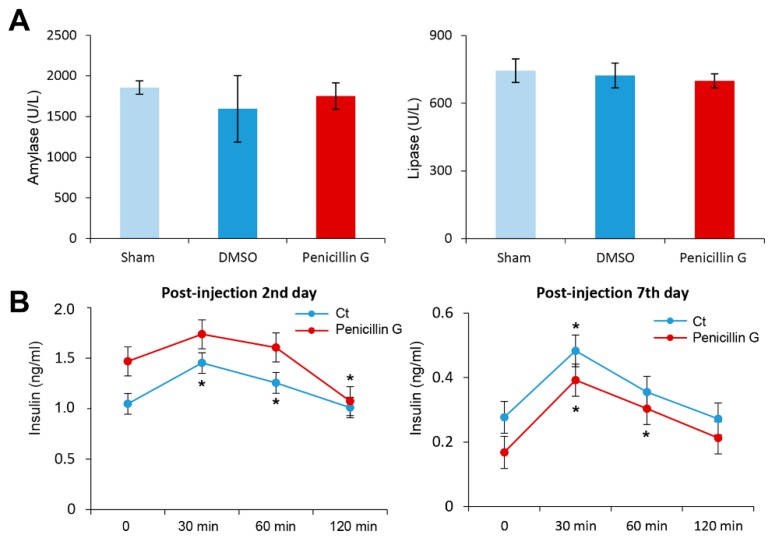
The effects of intrapancreatic penicillin G injection on pancreatic function. (**A**) Serum levels of amylase and lipase after intrapancreatic penicillin G injection. Injecting penicillin G into the pancreas did not significantly alter the serum levels of amylase or lipase. (**B**) Serial measurement of serum levels of insulin at certain time intervals by enzyme-linked immunosorbent assay (ELISA) after glucose infusion on the second and seventh day of penicillin G injection. Penicillin G-injected mice had slightly less insulin secretion than the control group although it was not statistically significant. Values are presented as mean ± standard deviation of three independent experiments. * *p* < 0.05. Abbreviation: DMSO, dimethyl sulfoxide.

We investigated whether pancreatic function is impaired by the injection of penicillin G into the pancreas. We first investigated the effect of penicillin G injection on the serum levels of amylase and lipase. Injecting penicillin G into the pancreas did not alter the serum levels of amylase or lipase significantly (Figure 4A).

Next, we examined whether the endocrine function of the pancreas is affected by the injection of penicillin G into the pancreas. In both the experiment and the control groups, we compared changes in serum insulin concentration at certain time intervals after intraperitoneal administration of 10% glucose (400 μL, 2 g/kg) on the second and seventh day of penicillin G injection, respectively (Figure 4B). Penicillin G-injected mice showed slightly less insulin secretion than the control group without statistical significance. Taken altogether, we could conclude that penicillin G did not impair exocrine and endocrine function of the pancreas.

### 2.6. Investigation of Mechanism Leading to Pancreatic Fibrosis by Penicillin G

**Figure 5:**
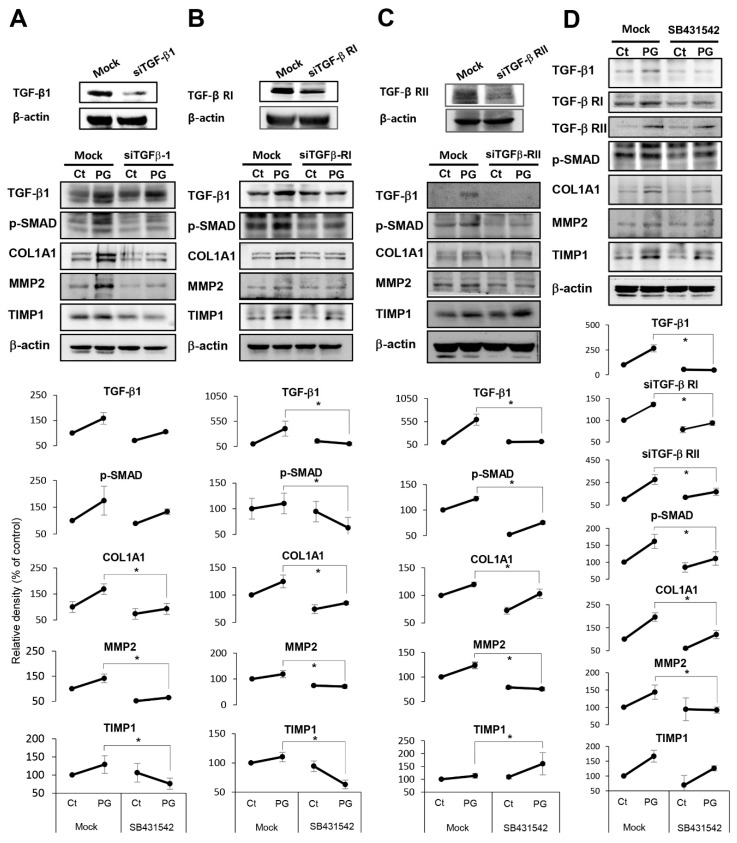
In vitro demonstration of mechanism leading to pancreatic fibrosis by penicillin G. (**A**) Western blot analysis of HPSCs after treating penicillin G followed by addition of TGF-β1 siRNA. The addition of TGF-β1 siRNA significantly abrogated profibrotic effects of penicillin G, demonstrated by the lower expression of profibrotic markers, such as collagen, MMP2, and p-SMAD. (**B**) Western blot analysis of HPSCs after treatment with penicillin G followed by addition of TGF-β receptor I (TGF-β RI) siRNA. The addition of TGF-β1 siRNA significantly abrogated profibrotic effects of penicillin G. (**C**) Western blot analysis of HPSCs after injecting penicillin G followed by addition of TGF-β receptor II (TGF-β RI) siRNA. The addition of TGF-β1 RI siRNA also significantly abrogated profibrotic effects of penicillin (**D**) Alterations in fibrosis-related factors after treatment of pancreatic tissue with Penicillin G combined with SB431542, an inhibitor of the TGF-β1 receptor. Treatment with SB431542 significantly abrogated the expression of all fibrosis-related factors that had been increased by Penicillin G. * *p* < 0.05.

TGF-β signaling plays an essential pathogenic role in a variety of fibrotic diseases. We thus performed in vitro experiments using HPSCs to determine at what levels does penicillin G block the TGF-β signaling pathway. We investigated the TGF-β signaling pathway at the levels of TGF-β1 agonist, TGF-β receptor I (TGF-β RI), and TGF-β receptor II (TGF-β RII), respectively, using the RNA interference approach. Western blot analysis showed that TGF-β1 siRNA significantly abrogated the profibrotic effects of penicillin G (*p* < 0.05) (Figure 5A). Specifically, the addition of TGF-β1 siRNA significantly abrogated the expression of fibrosis-related markers, such as collagen, MMP2, and phospho-SMAD (p-SMAD). Likewise, TGF-βR1 and TGF-βR2 siRNAs significantly abrogated profibrotic effects of penicillin G, suggesting that TGF-βR1 and TGF-βR2 are significantly involved in profibrotic effects of penicillin G (*p* < 0.05) (Figure 5B,C). Next, we examined that how SB431542, an inhibitor of the TGF-β1 receptor, alters the expression of fibrosis-related factors of the pancreas in the penicillin G-treated mice. Treatment with SB431542 significantly abrogated the expression of all fibrosis-related factors that had been increased by Penicillin G (*p* < 0.05) (Figure 5D). This result reaffirmed that penicillin G exerts a profibrotic effect through the TGF-β1 receptor.

**Figure 6:**
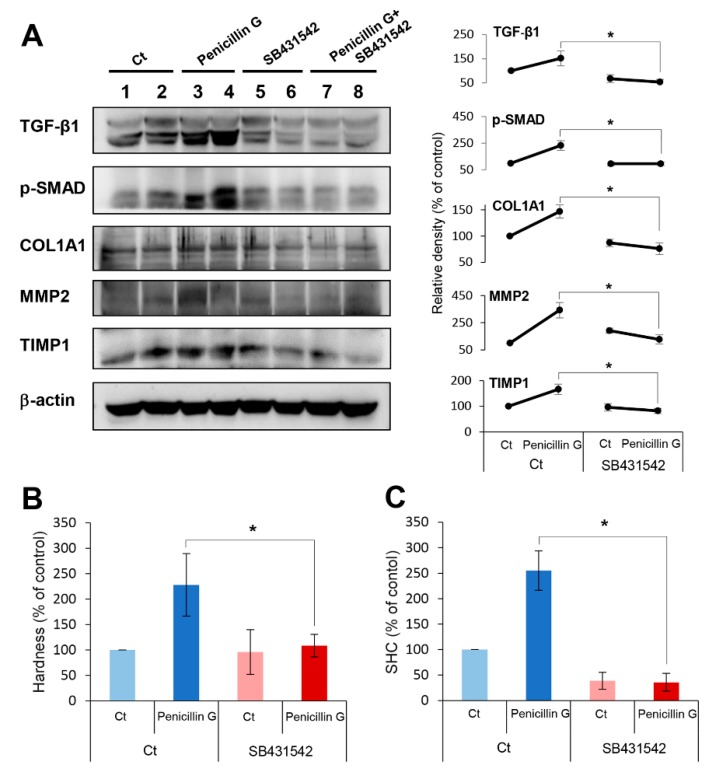
In vivo demonstration of mechanism leading to pancreatic fibrosis by penicillin G. Mice were injected with SB431542, an inhibitor of the TGF-β1 receptor intraperitoneally, followed by an intra-pancreatic injection of penicillin G. (**A**) Western blot analysis showing the expression of fibrosis-related markers in the mouse pancreas. Pretreatment with SB431542 in mice injected with intrapancreatic penicillin G significantly reduced the expression of fibrosis-related markers, such as TGF-β1, p-SMAD, collagen, MMP2, and TIMP1, in comparison with mice without the pretreatment (*p* < 0.05). (**B**) Measurement of pancreatic hardness. Pretreatment of SB431542 significantly abrogated the pancreatic hardening capacity by penicillin G in the mouse pancreas. (**C**) Measurement of SHC of the pancreas. Pretreatment of SB431542 significantly abrogated the enhanced SHC induced by penicillin G in the mouse pancreas. Values are presented as mean ± standard deviation of three independent experiments. * *p* < 0.05. Abbreviations: Ct, control; MMP-2, metalloproteinases-2; p-SMAD2, phosphorylated Smad2; SHC, suture holding capacity; TGF-β1, transforming growth factor-β1; TIMP-1, tissue inhibitor of metalloproteinases-1.

Subsequently, we performed in vivo experiments to determine profibrotic effects by penicillin G in the mouse pancreas. Mice were injected with SB431542, the inhibitor of the TGF-β1 receptor intraperitoneally, followed by an intra-pancreatic injection of penicillin G. Pretreatment of SB431542 to the mice injected with intrapancreatic penicillin G significantly reduced the expression of fibrosis-related markers, such as TGF-β1, p-SMAD, collagen, MMP2, and TIMP1, compared to the mice without the pretreatment (*p* < 0.05) (Figure 6A). Moreover, pretreatment of SB431542 significantly abrogated the pancreatic hardness and SHC increased by penicillin G in the mouse pancreas (*p* < 0.05) (Figure 6B,C). Taken altogether, these inhibition tests suggested that penicillin G enhances pancreatic hardness and SHC by upregulating pancreatic fibrosis that is mediated by TGF-β RI.

## 3. Discussion

In this study, we intended to provide a novel approach to prevent POPF. Screening tests for compounds from an antibiotics library revealed that the intrapancreatic injection of penicillin G significantly enhances the hardness and SHC of the pancreas. Subsequently, it was found that the intrapancreatic injection of Penicillin G activated HPSCs to produce various fibrotic materials such as α-SMA, MMP2, and TIMP1. Of note, penicillin G maximized pancreatic fibrosis at 2–3 days after injection, the time that is usually coinciding with the prevalence of POPF. Penicillin G induced reversible fibrosis, which does not cause permanent damage to pancreas and is normalized after seven days of injection. Also, we performed a wide variety of toxicity assays, and none of them demonstrated any significant toxicity associated to penicillin G. Accumulating evidence indicates that POPE is less likely to happen in the pancreas with higher stiffness and SHC. We thus believe that penicillin G is highly qualified for being an ideal profibrotic material that is capable of preventing POPF by enhancing pancreatic hardness and SHC.

In our study, although both penicillin G and ampicillin increased pancreatic hardness and SHC, they showed a difference in their manifestations. Ampicillin increased the hardness and SHC of the pancreas at specific concentrations, and gradually increased them over time. On the other hand, penicillin G increased the hardness and SHC dose-dependently, and increased them the most at the second day after injection. We believe that such differences are caused by discrepancies in the biochemical properties of these two compounds. Although Penicillin G and ampicillin both have a β-lactam ring and have similar molecular weights, they exhibit significant differences in protein binding, bioavailability, and metabolism [35]. Ampicillin has a 2-amino-2-phenylacetoamide group and a free acid, which has the same properties as zwitterion, while penicillin G has a phenylacetoamide group at position 6 from the β-lactam ring. Ampicillin has a high bioavailability of 30–55% and metabolism close to 50%, while penicillin G has a low bioavailability of 15–30% and is not well metabolized. In addition, ampicillin has a protein binding value of less than 25%, while penicillin G has a high protein binding of 60% [36]. Because of differences in biochemical characteristics, when each of these compounds is injected into the pancreas, penicillin G is expected to be attached to the proteins in the pancreatic tissue more strongly than ampicillin, resulting in having sufficient time to be reactive with HPSCs. These differences could lead to higher and more stable induction of pancreatic hardness and SHC by penicillin G in comparison with ampicillin.

Of all the risk factors for POPF, a soft consistency of the pancreatic parenchyma is one of the factors that is the most widely accepted [30,31,37,38,39,40,41]. Eschmuminov et al. [33] analyzed 22,376 patients included in 122 studies using the updated 2016 ISGPS (International Study Group Pancreatic Fistula) definition. They reported that the POPF rate was significantly higher in soft pancreas than in hard pancreas (RR, 4.4, 3.3 to 6,1; *p* < 0.001; *n* = 6393). Several reasons have been proposed to explain the higher frequency of POPF in soft pancreas. Firstly, soft pancreas is likely to have a pancreatic duct with an average diameter less than 3 mm [33]. Secondly, soft pancreas has a higher exocrine function than hard pancreas and thus may produce more enzyme-rich pancreatic juice [31,42]. Thirdly, soft pancreas is likely to have a relatively large number of side branches of the pancreatic duct [43]. However, the most crucial factor for soft pancreas to cause a high frequency of POPF is that soft pancreas has a poor SHC and thus tears easily during operation. Pancreatic operations accompany the manipulation of the pancreas, including the process of dividing pancreas and connecting it to the intestine, during which the pancreas tends to be disrupted due to its soft and fragile consistency, frequently leading to POPF.

The wound healing process is a natural process in which a damaged tissue is restored. If the wound healing process occurs at the appropriate time, the POPF can be minimized by it. Fibrosis is an essential component of the wound healing process [44,45]. The anastomotic site becomes compacted and adhesive as a result of fibrosis. However, the problem is that it is approximately 5–7 days after surgery when the pancreatico-enteric anastomotic site becomes adhesive and hardened by fibrosis. In other words, there is a gap between when the POPF is frequently encountered (POD 2–3) and when fibrosis is fully activated (POD 5–7). We expect penicillin G to play a role in reducing this gap by speeding up the fibrotic process. Wound healing process is composed of four essential steps; vascularization, inflammation, fibrosis, and resolution [44,45]. Since fibrosis is a prerequisite step for the wound healing process, “a rapid wound healing process” can be called “a faster fibrosis” in a broad sense. After pancreatic surgery, soft and fragile pancreas can easily cause POPF due to tearing. A rapid wound healing process is required to overcome this. The wound healing process of the pancreas results in the emptied spaces around the pancreatico-enteric anastomosis site stacked with compact fibrotic tissues, which can lower the chances of developing POPF.

Generally, POPF is first identified on 1–3 days after pancreatic surgery [46,47,48]. Our study showed that the time (POD 2–3) when POPF is peaked coincides with the time (POD 2–3) when penicillin G maximizes fibrogenesis in the pancreas. In addition, inducing “reversible” fibrosis is an essential prerequisite of the profibrogenic compounds aiming to prevent POPF, because persistent fibrosis can lead to pancreatic dysfunction and chronic pancreatitis. We validated that penicillin G did not induce prolonged fibrosis over seven days, when it is used within the proper concentration. We found that the pancreatic tissues returned to the pre-injection status in terms of hardness, SHC, function, and the degree of fibrosis, demonstrating its reversibility.

In conclusion, since a significant amount of POPF is closely related with soft pancreatic texture, we attempted to convert soft pancreatic texture into hard texture using chemical compounds. The intrapancreatic injection of Penicillin G activated PSCs to produce various fibrotic materials such as α-SMA, MMP2, and TIMP1, and led to an increase in the hardness and SHC of mouse pancreas. Interestingly, penicillin G maximized pancreatic fibrosis at 2–3 days after injection, usually coinciding with the time of onset of POPF. Moreover, penicillin G induced reversible fibrosis without causing permanent damage to the mouse pancreas. It is thus possible that penicillin is capable of preventing POPF by enhancing pancreatic hardness and SHC by enhancing pancreatic fibrosis. Our study, thus, suggests that penicillin G could be utilized for preventing or minimizing POPF, if its actual effectiveness and safety are validated by further studies.

## 4. Materials and Methods

### 4.1. Animals Study

Five-week male BALB/c mice (Orient Bio, Seongnam, Korea) were used in this study. Animal studies were carried out in compliance with the guidelines of the Institute for Laboratory Animal Research, Korea (IRB No: CMCDJ-AP-2017-001, 22 February 2017). The mice were allowed to acclimatize to their new environment for 14 days and were maintained in an environment with temperature 23 ± 3 °C, humidity 50 ± 10%, 12-h light–dark cycle with 150–300 lux, and ventilation at 10–20 times/hour. In this study, we performed three sets of animal experiments.

The first set (*n* =100) is a comparison of the effects on the mouse pancreas between penicillin G and ampicillin. We injected ampicillin and penicillin G into the pancreas with 5 mice per group (*n* = 50) at concentrations of 0, 0.1, 1.0, 10, and 100 mM of each compound, respectively, and measured the hardness and SHC of the pancreas after 48 h. Subsequently, we injected 1 mM ampicillin and 1mM penicillin G into the pancreas, respectively (*n* = 50), and measured the hardness and SHC of the pancreas after 0-day (*n* = 5), 1-day (*n* = 5), 2-day (*n* = 5), 3-day (*n* = 5), 5-day (*n* = 5), and 7-day (*n* = 5) after injection, respectively. Pancreatic hardness and SHC were measured using the digital force gauge (Shimpo instruments, NY, USA). Specifically, pancreatic hardness was measured in the manner of compressing the extracted pancreas with the instrument. Next, for the determination of pancreatic SHC, the suture that stitched the pancreas was connected to the instrument. Pancreatic SHC was determined by measuring the tensile strength exerted to the pancreatic tissue until it got torn.

The second set (N = 30) is the further characterization of the effects of penicillin G on the pancreas. We divided the total 30 mice into control (*n* = 5) and experimental (*n* = 25) groups, respectively. Control and experimental groups were received intrapancreatic injection of 1mM saline and 1mM penicillin G, respectively. Experimental groups were divided into five groups based on the days of measuring the hardness and SHC of the pancreas: 1-day (*n* = 5), 2-day (*n* = 5), 3-day (*n* = 5), 5-day (*n* = 5), and 7-day (*n* = 5) groups. Specifically, we performed laparotomy and identified the mouse pancreas under general anesthesia. We injected 1 mM penicillin G directly into the pancreatic neck portion to the mice in the experimental groups. At the determined dates, we collected the serum samples for the analysis. Subsequently, we extracted the mouse pancreas and measured pancreatic hardness and SHC using a digital force gauge.

The third set (N = 40) is TGF-β1 receptor inhibition test for determining the mechanism of penicillin G. We injected SB431542 (TGF-β1 receptor inhibitor) that had been dissolved in 5% DMSO to mice intraperitoneally at 10 mg/kg for 3 times for 2 weeks. Subsequently, penicillin G was administered of 0.1 mM/100 μL to the mouse pancreas, and the hardness and SHC of the pancreas were measured after 48 h. Experimental group were divided into four groups according to the treatment modality: control (*n* = 10), penicillin G (*n* = 10), SB431542 (*n* = 10), and SB431542 + Penicillin G (*n* = 10).

### 4.2. Statistical Analysis

All data were analyzed using SPSS 11.0 software (SPSS, Chicago, IL, USA) and are presented as the mean ± SD. The Mann–Whitney U-test was used for the mean comparison of two groups, and the Kruskal–Wallis test was used for the comparison of three or more groups. Probability (*p*) values of < 0.05 were considered statistically significant.

### 4.3. Additional Materials and Methods

Additional and more detailed information regarding the experimental procedures are fully described in the additional file.

## Abbreviations

α-SMA	alpha smooth muscle actin
COL1A1	collagen type 1 alpha1
DMSO	dimethyl sulfoxide
HPSCs	human pancreatic stellate cells
IL-6	interleukin-6
MMP2	metalloproteinases-2
POD	postoperative day
POPF	postoperative pancreatic fistula
ELISA	enzyme-linked immunosorbent assay
p-SMAD	pospho-SMAD
SHC	suture holding capacity
TGFβ-1	transforming growth factor-β1
TIMP1	tissue inhibitor of metalloproteinases-1
TNF-α	tumor necrosis factor- α
Ct	control
